# DSnet: a new dual-branch network for hippocampus subfield segmentation

**DOI:** 10.1038/s41598-024-66415-0

**Published:** 2024-07-03

**Authors:** Hancan Zhu, Wangang Cheng, Keli Hu, Guanghua He

**Affiliations:** 1 School of Mathematics, Physics and Information, Shaoxing University, 900 ChengNan Rd, Shaoxing, 312000 Zhejiang China; 2 Institute of Artificial Intelligence, Shaoxing University, Shaoxing, 312000 Zhejiang China

**Keywords:** Hippocampal subfield segmentation, Deep learning, Dual-branch network, U-Net, Image processing, Machine learning

## Abstract

The hippocampus is a critical component of the brain and is associated with many neurological disorders. It can be further subdivided into several subfields, and accurate segmentation of these subfields is of great significance for diagnosis and research. However, the structures of hippocampal subfields are irregular and have complex boundaries, and their voxel values are close to surrounding brain tissues, making the segmentation task highly challenging. Currently, many automatic segmentation tools exist for hippocampal subfield segmentation, but they suffer from high time costs and low segmentation accuracy. In this paper, we propose a new dual-branch segmentation network structure (DSnet) based on deep learning for hippocampal subfield segmentation. While traditional convolutional neural network-based methods are effective in capturing hierarchical structures, they struggle to establish long-term dependencies. The DSnet integrates the Transformer architecture and a hybrid attention mechanism, enhancing the network’s global perceptual capabilities. Moreover, the dual-branch structure of DSnet leverages the segmentation results of the hippocampal region to facilitate the segmentation of its subfields. We validate the efficacy of our algorithm on the public Kulaga-Yoskovitz dataset. Experimental results indicate that our method is more effective in segmenting hippocampal subfields than conventional single-branch network structures. Compared to the classic 3D U-Net, our proposed DSnet improves the average Dice accuracy of hippocampal subfield segmentation by 0.57%.

## Introduction

The hippocampus is an essential component of the brain, located on the medial side of the human temporal lobe, and it exists bilaterally, with one hippocampus in each hemisphere. It plays a pivotal role in memory, learning, spatial navigation, and emotional regulation. Numerous clinical studies have shown that the structure of the hippocampus is closely related to various brain diseases, including Alzheimer’s Disease (AD)^1^, Mild Cognitive Impairment (MCI)^2^, and depression^3^. The hippocampus can typically be further subdivided into several subfields. Atrophy in different hippocampal subfields can lead to various diseases. For instance, atrophy in the hippocampal head might result in schizophrenia, while shrinkage in the hippocampal tail could lead to depression^4^. Quantitative analysis of the morphological structure of hippocampal subfields aids in guiding the diagnosis of related diseases. Therefore, accurately segmenting hippocampal subfields from brain magnetic resonance images (MRI) holds significant practical importance^5,6^.

However, the structure of hippocampal subfields is irregular, and their boundaries are relatively ambiguous and complex. Their voxel values are close to surrounding brain tissues (such as white matter and amygdala), making the task of segmenting hippocampal subfields based on MRI quite challenging. Manual segmentation of the hippocampus has been a commonly utilized method; however, it depends on the expertise of medical professionals and is notably time-intensive. Consequently, the imperative for automating the segmentation of hippocampal subfields has become evident. Over the last two decades, a plethora of automatic segmentation techniques have emerged and been employed for precisely delineating hippocampal subfields. These mainly include multi-atlas based segmentation methods^7–11^ and deep learning based segmentation methods^12–20^.

Multi-atlas based segmentation methods commence by aligning the target image with a set of multiple atlas images. Following this alignment, the labels from these atlas images are projected onto the target image. Ultimately, a process of label fusion is executed to derive the definitive segmentation result^21–23^. Nonetheless, the effectiveness of this approach hinges on several critical factors, including the quality and quantity of available atlases, the precision of image registration, and the selection of an apt atlas fusion strategy. Given the intricate nature of hippocampal subfields and their subtle grayscale distinctions, conventional multi-atlas segmentation techniques often fall short in accurately delineating these subfields. Yushkevich and colleagues enhanced the classical multi-atlas segmentation method by refining the approach for weight calculation and incorporating the Adaboost classification algorithm to rectify errors in the segmentation of hippocampal subfields^7^. Wang and their research team introduced a globally optimized segmentation approach that, through the imposition of constraints on the similarity between pairs of atlases, successfully mitigated errors stemming from the overlap of similar atlas displacement fields^8^. In response to the challenge of limited data, Jon Piptone and their collaborators introduced the MAGeT (Multiple Automatically Generated Templates) method. This innovative approach employs multi-atlas registration to generate multiple templates and subsequently employs multi-atlas segmentation with these templates as a basis^9^. Within the context of multi-atlas segmentation, Zhu and their research team harnessed the power of deep learning techniques to rectify label errors stemming from image registration, thereby significantly augmenting the overall effectiveness of the multi-atlas segmentation approach^24^.

Deep convolutional neural networks (CNNs) initially made significant strides in domains such as image classification and object detection, and they have now become a prevalent choice for the task of medical image segmentation. In contrast to conventional CNNs, the Fully Convolutional Network (FCN)^18^ replaces fully connected layers with convolutional layers. This transformation equips the network to process images of variable scales, thereby boosting its adaptability and computational efficiency. Expanding upon the FCN framework, Zhu and colleagues introduced DU-Net, a novel architecture that incorporates dilated dense networks into the U-Net structure, enabling the extraction of multi-scale features at high resolutions. Moreover, they innovatively replaced the conventional convolutional blocks with residual connections, giving rise to ResDU-Net, which streamlines and enhances the process of feature integration, further boosting its effectiveness^13^. Khalili and his colleagues introduced the Dense-Dense U-Net, a sophisticated multi-scale network architecture meticulously crafted for the precise segmentation of the amygdala and hippocampus^19^. This network adaptively captures a fusion of both local and global information, resulting in the extraction of more nuanced and intricate features. In a related development, Hung and colleagues introduced a hierarchical feedback-connected network, which integrates a hierarchical structure and a feedback mechanism. This unique network architecture excels at capturing the hierarchical structure and dependencies within the data more effectively^20^. A noteworthy challenge in conventional convolutional neural networks lies in their struggle to establish long-distance dependencies, which imposes limitations on their capacity for comprehensive image feature processing.

The Transformer architecture, known for its proficiency in modeling global contexts within images, exhibits formidable feature processing capabilities. In recent years, it has found notable success in the domain of medical image segmentation^25–30^. Xie et al. introduced the variable self-attention mechanism within the CoTr network, enabling a laser focus on specific regions of the feature map. This innovation effectively reduces computational overhead and algorithmic complexity^29^. Wang and collaborators innovatively embedded the Transformer architecture into the U-Net framework, creating the TransBTS structure. This integration enhances the network’s ability to grasp global semantic information, thereby expanding its receptive field^30^. Ranem and their team presented a Transformer-based ViT architecture tailored for hippocampal segmentation, underscoring the continuous learning prowess of Transformers in the realm of medical imaging^31^. Liu et al. proposed PHTrans, a novel approach that blends CNNs with Transformers, allowing for the simultaneous acquisition of local and global features. This harmonious combination harnesses the inherent strengths of both paradigms^32^.

In this research, we introduce a novel dual-branch U-shaped network architecture, DSnet, meticulously tailored for the segmentation of hippocampal subfields. This innovative design comprises two distinct branches: one dedicated to segmenting the hippocampus as a whole, and the other laser-focused on delineating its individual subfields. We employ residual connections to seamlessly merge the insights from the hippocampal segmentation branch into the subfield segmentation, thus providing crucial guidance for the accurate delineation of hippocampal subfields. The overarching network architecture includes an encoder, a decoder, a Transformer network, and strategically positioned skip connections. Both the encoder and decoder harness the power of convolutional neural networks to extract essential low-level features, while the Transformer network employs self-attention mechanisms to capture extensive global image information. Within the skip connections, we introduce a hybrid attention mechanism that adaptively blends spatial and channel attention strategies. This adaptive selection of meaningful features empowers the model to focus sharply on critical image regions while disregarding irrelevant areas, thereby enhancing segmentation precision^33^.

The main contributions of this article can be summarized as follows:

1. Innovative Dual-Branch Network: We introduce a pioneering dual-branch network architecture named DSnet, meticulously crafted for the precise segmentation of hippocampal subfields.
2. Enhanced Feature Extraction: Our proposed network seamlessly integrates the Transformer structure and a hybrid attention mechanism, elevating the network’s global perceptual capabilities. Consequently, it significantly enhances the network’s feature extraction performance.
3. Rigorous Validation: To validate our model, we conducted experiments using the publicly available Kulaga-Yoskovitz dataset. The results unmistakably demonstrate that our method outperforms current mainstream network structures in terms of accuracy.

## Method

**Figure 1:**
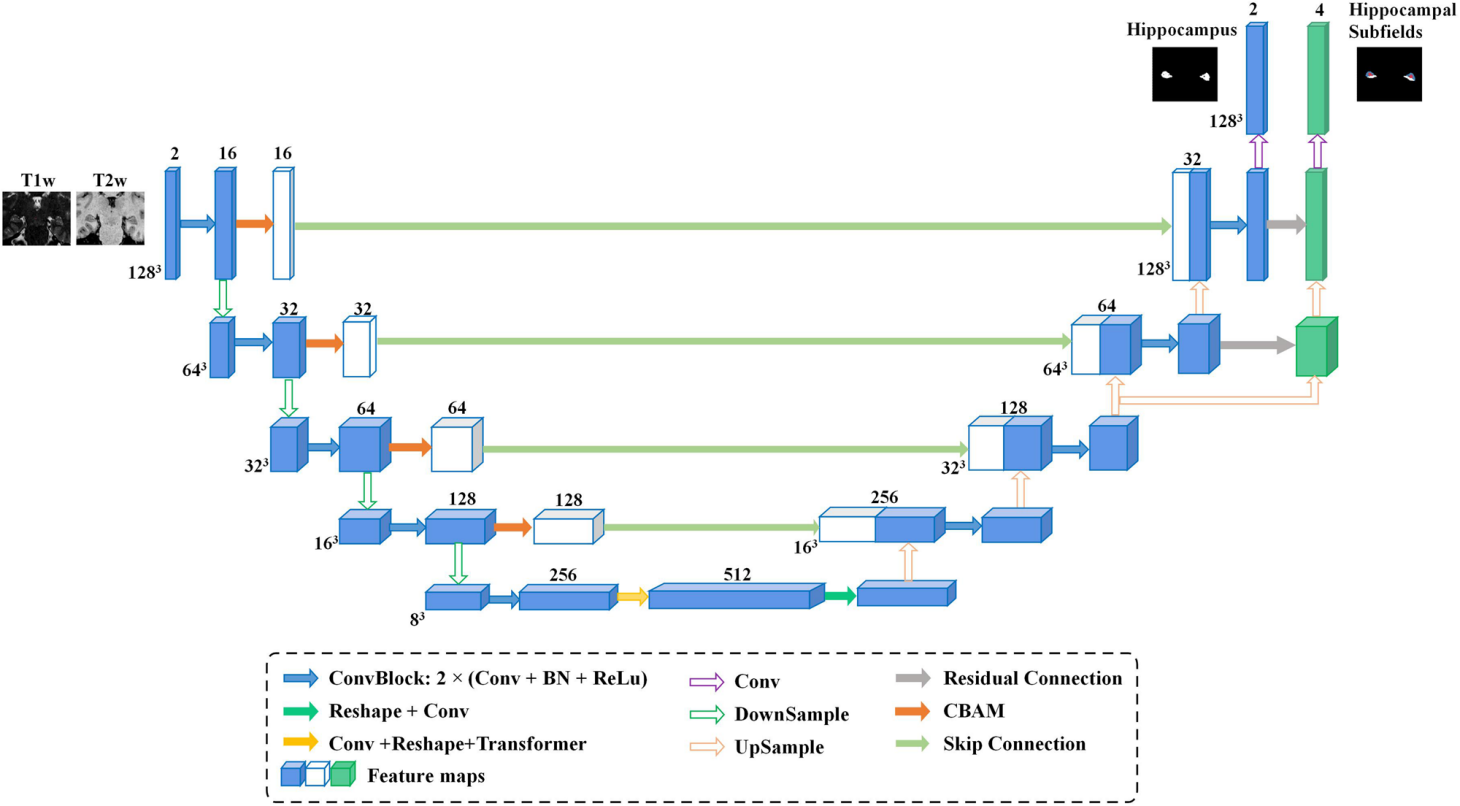
Overview of the DSnet architecture. It contains a convolutional encoder, a Transformer block embedded at the bottom, long-connection modules equipped with a Convolutional Block Attention Module (CBAM), and a multi-branch decoder.

In this research, we propose DSnet, a purpose-built network tailored for the precise segmentation of hippocampal subfields. Drawing inspiration from the U-Net^34^ architecture, DSnet incorporates key elements such as a convolutional encoder, a Transformer network, long-connection modules equipped with a hybrid attention mechanism, and a multi-branch decoder. In DSnet, we apply the Transformer to the bottleneck layer of U-Net. The image features in the bottleneck layer have undergone multiple convolutions and contain rich high-level information, making them more suitable for global context interaction and extraction using the Transformer. Figure 1 provides an overview of the comprehensive DSnet framework, and in the subsequent sections, we will delve into the intricate details of each module.

### Convolutional encoder

The convolutional encoder within DSnet closely mirrors the structure found in U-Net, featuring 5 ConvBlocks and 4 pooling layers. Each ConvBlock consists of a sequential combination of components, including a convolutional layer, a batch normalization layer, and a ReLU activation layer. Notably, the convolutional layer employs a 3 × 3 × 3 kernel size. For the pooling layers, we employ a max-pooling operation with both a kernel size and stride of 2 to facilitate down-sampling of the feature maps. The output feature map following each ConvBlock can be denoted as $F_{l}^{local} \in {\mathbb{R}}^{{C \times \frac{D}{{2^{l - 1} }} \times \frac{H}{{2^{l - 1} }} \times \frac{W}{{2^{l - 1} }}}}$, where $l$ signifies the encoding stage ($l$ = 1, 2, 3, 4, 5); $D$, $H$, and $W$ represent the depth, height, and width of the input image block, respectively; and $C$ indicates the channel number, which is set at $16 \times 2^{{\left( {l - 1} \right)}}$ in this context.

### Transformer

**Figure 2:**
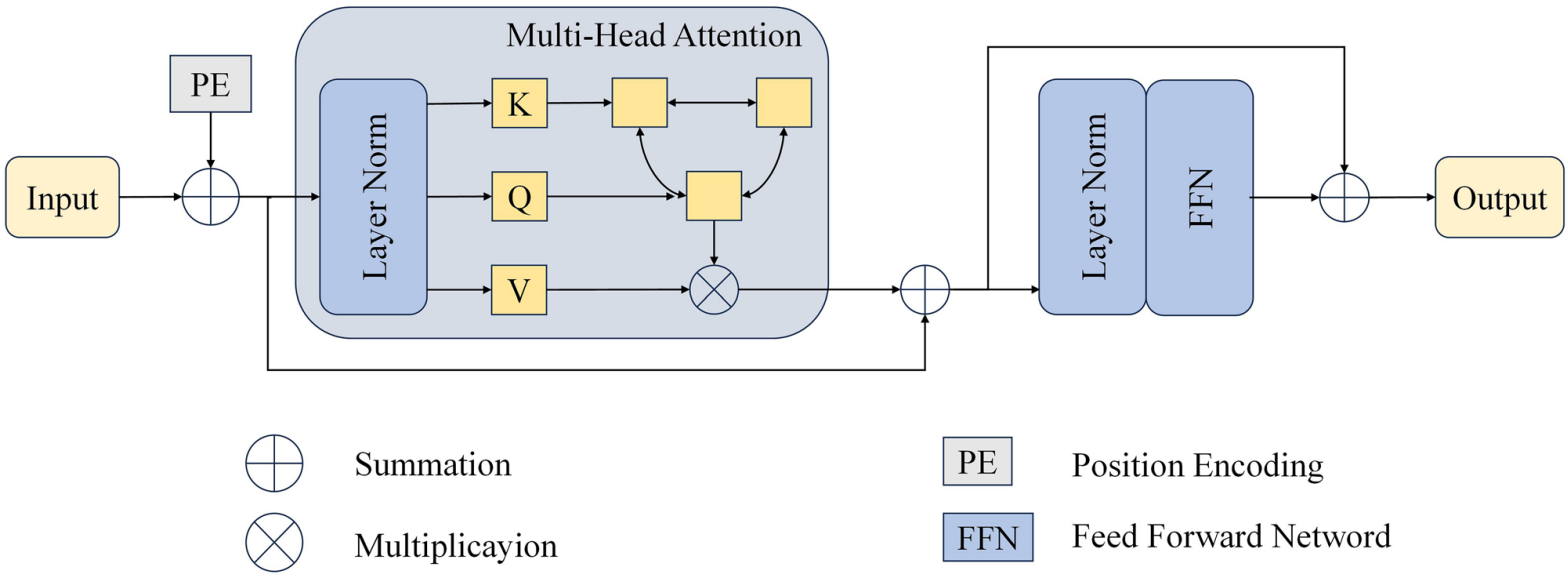
Diagram illustrating the transformer architecture.

Recognizing the challenge that convolutional networks face in establishing long-range dependencies, we have integrated a Transformer structure at the bottom of the encoder for more effective contextual feature extraction. This Transformer architecture, depicted in Fig. 2, encompasses three essential components: a Tokenizer, a Multi-Head Self Attention (MSA), and a Feed-Forward Network (FFN).

When embedding the Transformer in a Sequence-to-Sequence fashion, the local feature map $F_{5}^{local}$ is transformed into a 1D sequence and linearly projected into the token space. However, this flattening operation inevitably results in the loss of spatial information. To address this, we introduce learnable positional embeddings denoted as $P$, which are added to the tokens through element-wise summation. The formula is as follows:

$$ F^{token} = F_{5}^{local} \;W + P. $$

Here, $F^{token} \in R^{{C^{\prime} \times \frac{DHW}{{2^{{3\left( {l - 1} \right)}} }}}}$ represents the tokens, and $W$ denotes the weights for the linear projection. The calculation for the Multi-Head Self Attention (MSA) operator is expressed as:

$$ head^{i} = Attention\left( {Q_{i} ,\;K_{i} ,\;V_{i} } \right) = soft\max \left( {\frac{{Q_{i} K_{i}^{T} }}{{\sqrt {d_{k} } }}} \right)V_{i} , $$

$$ MSA = \left[ {head^{1} , \ldots ,head^{N} } \right]W^{O} . $$

In this context, $Q_{i} = LN\left( {F^{token} } \right)W^{{Q_{i} }}$, $K_{i} = LN\left( {F^{token} } \right)W^{{K_{i} }}$, $V_{i} = LN\left( {F^{token} } \right)W^{{V_{i} }}$, where $LN\left( \cdot \right)$ signifies layer normalization, $d_{k}$ represents the dimension of $K$, $N = 8$ indicates the number of heads in the self-attention mechanism, and $\left[ { \cdot , \cdot } \right]$ denotes the concatenation operation. The weights $W^{{Q_{i} }} ,\;W^{{K_{i} }} ,\;W^{{V_{i} }} ,$ and $W^{O}$ are all adjustable parameters. The Feed-Forward Network (FFN) is a two-layer perceptron activated by GELU^35^. The feature map incorporating global contextual information generated by this Transformer structure is represented as:

$$ y = MSA\left( {LN\left( {F^{token} } \right)} \right) + F^{token} , $$

$$ F^{global} = FFN\left( {LN\left( y \right)} \right) + y, $$

where $F^{global} \in {\mathbb{R}}^{{C^{\prime} \times \frac{DHW}{{2^{{3\left( {l - 1} \right)}} }}}} .$ This formulation ensures that the Transformer structure captures global contextual information, thereby enhancing the overall model performance.

### Convolutional block attention module

**Figure 3:**
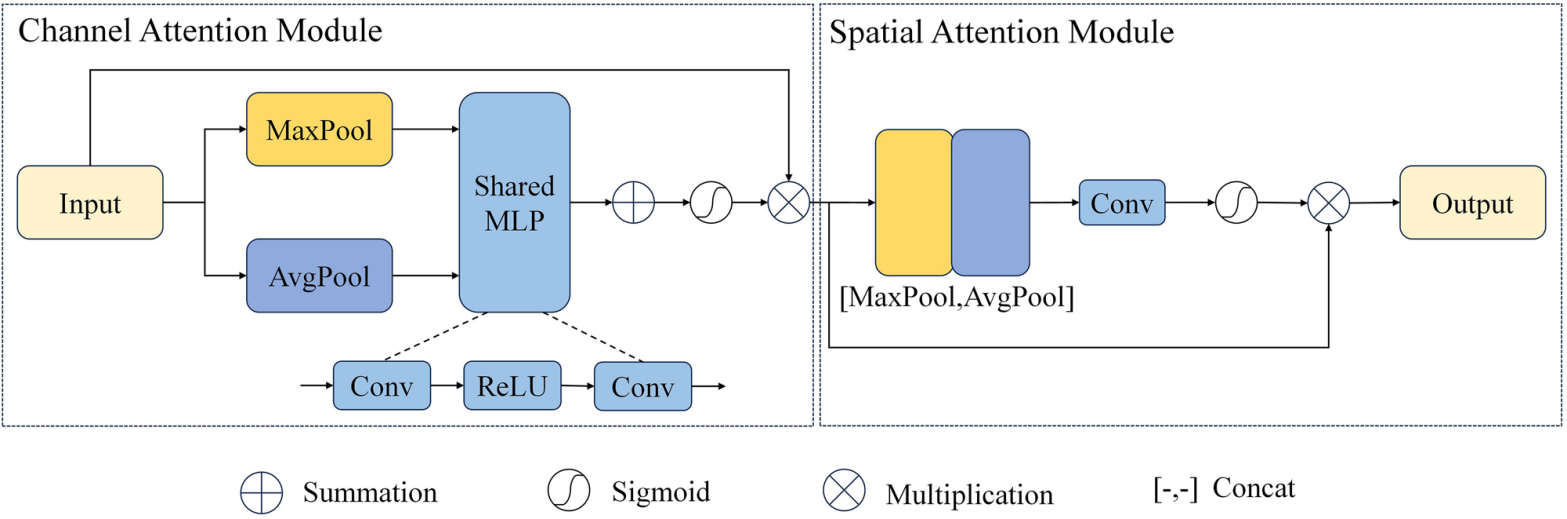
Diagram illustrating the CBAM module. It contains a channel attention module and a spatial attention module. “MLP” represents Multi-Layer Perceptron.

The Convolutional Block Attention Module (CBAM) was initially introduced by Woo et al.^33^. In our adaptation for 3D image data segmentation, we have made slight adjustments, specifically by substituting the original 2D convolutions with 3D convolutions. The CBAM module’s architecture, as depicted in Fig. 3, primarily consists of two key components: channel attention and spatial attention.

In the channel attention mechanism, we leverage both average pooling and max pooling. Average pooling captures the global context, while max pooling focuses on local features. Employing both simultaneously enhances the network’s robustness. Both pooling pathways share a Multi-Layer Perceptron (MLP). The results from these pathways are summed up, and after applying a Sigmoid activation function, the output is element-wise multiplied with the input feature map of the module. This process is represented by the following formula:

$$ M_{c} \left( {F_{l}^{local} } \right) = \sigma \left( {MLP\left( {Maxpool\left( {F_{l}^{local} } \right)} \right) + MLP\left( {Avgpool\left( {F_{l}^{local} } \right)} \right)} \right) \otimes F_{l}^{local} . $$

Here, $M_{c} \left( {F_{l}^{local} } \right)$ with dimensions ${\mathbb{R}}^{{C \times \frac{D}{{2^{i - 1} }} \times \frac{H}{{2^{i - 1} }} \times \frac{W}{{2^{i - 1} }}}}$ represents the output of the channel attention at layer $l$. The symbol ${\upsigma }$ denotes the Sigmoid activation function, and we denote the modified feature map as $F^{\prime} = M_{c} \left( {F_{l}^{local} } \right).$

The output from the channel attention mechanism serves as the input to the spatial attention mechanism. Here, we also apply both max pooling and average pooling to this input, generating two feature maps. These feature maps are then concatenated and subjected to a convolution operation with a kernel size of 7 × 7 × 7. The final output, following a Sigmoid activation function, is element-wise multiplied with the feature map from the module. This process is represented by the formula:

$$ M_{s} \left( {F^{\prime}} \right) = \sigma \left( {Conv\left( {\left[ {MaxPool\left( {F^{\prime}} \right),\;AvgPool\left( {F^{\prime}} \right)} \right]} \right)} \right) \otimes F^{\prime}, $$

where $M_{s} \left( {F^{\prime}} \right)$ with dimensions ${\mathbb{R}}^{{C \times \frac{D}{{2^{i - 1} }} \times \frac{H}{{2^{i - 1} }} \times \frac{W}{{2^{i - 1} }}}}$ signifies the output of the spatial attention mechanism.

### Dual-branch decoder

Our decoder architecture closely resembles the U-Net design^34^. We employ upsampling via a deconvolution operation with a kernel size of 2 and a stride of 2, followed by a ConvBlock that aligns with the encoder for efficient feature extraction. After applying two upsampling operations, we partition the network into two branches. One branch is dedicated to the holistic segmentation of the entire hippocampus, while the other branch is purpose-built for segmenting the specific subfields within the hippocampus. These two branches are seamlessly connected through residual connections^36^, where the feature map from the overall hippocampus segmentation branch is combined with the feature map of the subfield segmentation branch. This strategic integration guides the segmentation of hippocampal subfields using the broader segmentation context of the entire hippocampus.

## Experiments and results

### Dataset and preprocessing

We used the publicly available Kulaga-Yoskovitz dataset to validate our method (https://www.nitrc.org/projects/mni-hisub25)^37^. This dataset includes T1-weighted (T1w) and T2-weighted (T2w) brain images from 25 subjects, as well as segmentation labels for hippocampal subfields such as CA1-3, CA4/DG, and Sub. The T1w image is isotropic with a resolution of 0.6 × 0.6 × 0.6 mm^3^, while the T2w image is anisotropic with a resolution of 0.4 × 0.4 × 2 mm^3^. Before our analysis, the dataset underwent a series of detailed image preprocessing steps. These steps included automated intensity inhomogeneity correction and intensity normalization for all T1w and T2w images, linear registration of all images to the MNI152 standard space, and resampling to a consistent resolution of 0.4 × 0.4 × 0.4 mm^3^. To reduce interpolation artifacts in the images, the upsampling process utilized the nonlocal super-resolution method^38^. For more details, please refer to the original paper^37^.

Due to the relatively fixed position of the hippocampal structure in the brain, we determined the minimum and maximum coordinates of the hippocampal region by scanning all training data. We then expanded this region by adding 32 voxels in each direction to form a bounding box, ensuring it could cover the hippocampal region in external data. For particularly abnormal brains, the bounding box needs to be manually determined. Using this bounding box, we cropped the hippocampal region from all images, resizing them to a uniform size of 267 × 182 × 174. Subsequently, we corrected the image intensities through histogram matching. To maximize the use of limited data, we horizontally flipped each training image, effectively doubling the size of our training dataset.

### Training and inference details

We conducted our experiments using the PyTorch framework, leveraging the power of two NVIDIA GeForce RTX 3090 Ti GPUs. The neural network was trained using the Adam optimizer and employed the poly learning rate scheduling strategy. Our initial learning rate was set at 0.001, with a decay factor of 0.9 applied after each iteration. Throughout the training process, we used a batch size of 4 to optimize performance. In order to enhance the model’s input, we applied random cropping, extracting image blocks measuring 128 × 128 × 128. Additionally, we implemented several image augmentation techniques, including random mirroring in axial, coronal, and sagittal orientations with a probability of 0.5. To introduce variability in voxel intensities, we allowed for random intensity shifts within the range of [− 0.1, 0.1]. Moreover, we performed random scaling of images within the [0.9, 1.1] range to further augment the dataset. For the network’s loss function, we adopted the Dice loss^39^, which is defined as:

$$ {\mathcal{L}}\left( {Y,\;\tilde{Y}} \right) = - \frac{1}{N}\mathop \sum \limits_{n = 1}^{N} \frac{{2Y_{n} \widetilde{{Y_{n} }}}}{{Y_{n} + \widetilde{{Y_{n} }}}}. $$

Here, $Y_{n}$ and $\widetilde{{Y_{n} }}$ represent the ground truth and predicted probability, respectively, while $N$ is the batch size. The total loss of the network is further defined as:

$$ {\mathcal{L}}_{total} = \lambda_{1} {\mathcal{L}}_{1} + \lambda_{2} {\mathcal{L}}_{2} , $$

where ${\mathcal{L}}_{1}$ and ${\mathcal{L}}_{2}$ denote the losses for the binary (hippocampus) and multi-class (hippocampal subfield) branches, respectively, and we set $\lambda_{1} = \lambda_{2} = 0.5$. For model regularization, we used the L2 norm with a weight decay rate of $10^{ - 5}$. The network training was terminated after 8000 epochs.

**Figure 4:**
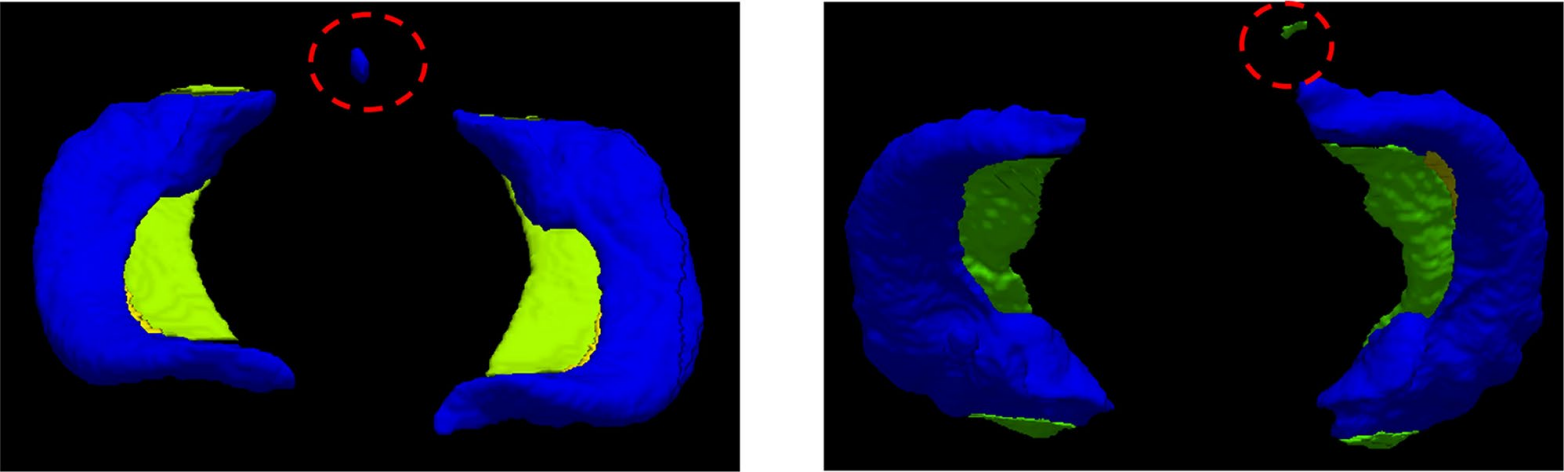
Examples of false positives in DSnet segmentation results before post-processing.

During the testing phase, we employed a non-overlapping sliding window strategy to extract image blocks of size 128 × 128 × 128, which were then input into the model for segmentation. The inference results were obtained by averaging the outputs of the model from the last four epochs. Due to the presence of false positives in the model’s segmentation results, as shown in Fig. 4, we proposed a post-processing method to eliminate these false positives. We generated a binary mask to identify all connected target regions and empirically set a threshold of 1000 voxels, which corresponds to a size of 64 mm^3^. Any connected region smaller than this threshold was considered a non-hippocampal region and thus labeled as background. This method helps mitigate model errors and reduce noise, thereby improving the overall segmentation performance.

To evaluate the performance, we selected the Dice coefficient and the 95% Hausdorff distance (HD95) as evaluation metrics. The Dice coefficient quantifies the overlap between the automated segmentation and the precise manual annotations, providing a measure of segmentation accuracy. It is defined as follows:

$$ Dice = 2\frac{V(A \cap B)}{{V(A) + V(B)}}, $$

where $A$ represents the precise annotation, $B$ represents the network’s automatic segmentation, and *V*(*S*) denotes the volume of $S$. The Hausdorff Distance 95% is a robust version of the Hausdorff distance, assessing the robustness of the segmented structure and the consistency of the segmentation boundaries. The Hausdorff distance is defined as:

$$ H\left( {A,B} \right) = \max \left( {h\left( {A,B} \right),h\left( {B,A} \right)} \right), $$

where $h\left( {A,B} \right) = \mathop {\max }\limits_{a \in A} \;\mathop {\min }\limits_{b \in B} \;d\left( {a,b} \right)$, and $d\left( { \cdot , \cdot } \right)$ represents the Euclidean distance. After computing all the distances, they were arranged in ascending order, and the distance corresponding to the 95th percentile was designated as HD95.

Our experiments were conducted using a five-fold cross-validation approach. Within each fold, we allocated 15 participants for training, 5 participants for validation, and an additional 5 participants for testing. In this manner, all participants serve as the test set in one of the folds, and we report the segmentation accuracy for all participants.

### Comparison with other segmentation networks

**Figure 5:**
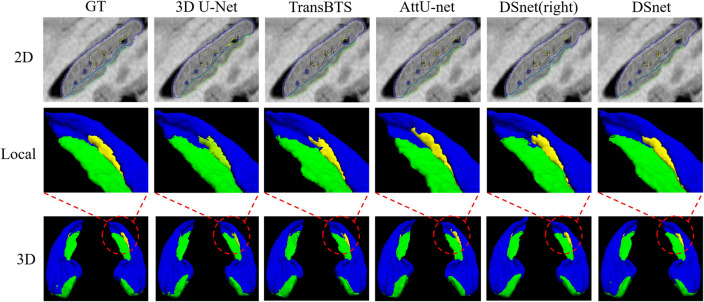
Segmentation results for a randomly selected subject: 2D segmentations (top row), 3D segmentations (bottom row), and 3D Close-Ups (middle row). Blue: CA1-3, Green: CA4/DG, Yellow: SUB. “GT” represents Ground truth and “DSnet (right)” refers to the exclusive training of the right hippocampal subfield segmentation branch of DSnet.

We conducted a comparative analysis of our proposed method against well-established networks known for their exceptional segmentation prowess, namely, the 3D U-Net^40^, TransBTS^30^, and AttentionU-Net (AttU-Net)^41^. The comprehensive experimental results are available in both Tables 1 and 2. These findings demonstrate our DSnet performs comparably to mainstream state-of-the-art segmentation techniques. On average, DSnet outperforms other networks with a Dice coefficient that is 0.57% higher than that of the 3D U-Net and an average HD95 score that is 0.14 lower than the 3D U-Net. Additionally, we conducted a statistical test using the Wilcoxon Signed-Rank Test. The results show that for the Dice metric, the DSnet method is significantly superior to other methods. However, for the HD95 metric, the DSnet method did not show statistical significance, indicating that the boundary accuracy of the proposed method is comparable to other methods and requires further investigation or optimization to achieve significant improvements. To provide further insight, visualized segmentation results are showcased in Fig. 5.

**Table 1:** Dice scores of segmentation results on the Kulaga-Yoskovitz dataset. A higher Dice value signifies superior segmentation performance, with the top-performing results showcased in bold. Statistical analysis was conducted using the Wilcoxon Signed-Rank Test to compare each model with DSnet, where a p-value < 0.05 indicates a significant difference between the two methods. It is worth noting that “DSnet (right)” refers to the exclusive training of the right hippocampal subfield segmentation branch of DSnet. Significant values are in italics.

		CA1-3	CA4/DG	SUB	Hippocampus
3D U-Net^40^	Mean (std)	91.08 (1.6)	88.44 (1.9)	87.35 (2.1)	95.38 (1.0)
	p-value	*5.3e−4*	*3.4e−2*	*1.3e−2*	*8.7e−3*
TransBTS^30^	Mean (std)	91.26 (1.2)	88.61 (1.7)	86.75 (2.0)	95.64 (0.7)
	p-value	*8.2e−5*	*2.6e−3*	*8.0e−5*	*6.1e−2*
AttU-Net^41^	Mean (std)	91.24 (1.2)	88.39 (1.9)	87.22 (2.0)	95.58 (0.7)
	p-value	*3.4e−3*	*3.9e−3*	*2.0e−4*	*6.0e−3*
DSnet (right)	Mean (std)	91.40 (1.2)	88.42 (1.9)	87.68 (1.9)	95.56 (0.6)
	p-value	*1.2e−3*	*3.2e−2*	*6.2e−2*	*2.3e−3*
DSnet	Mean (std)	**91.68 (1.2)**	**88.79 (1.6)**	**88.10 **(2.0)	**95.75 (0.6)**

**Table 2:** HD95 scores of segmentation results on the Kulaga-Yoskovitz dataset. A lower HD95 value signifies superior segmentation performance, with the top-performing results showcased in bold. Statistical analysis was conducted using the Wilcoxon Signed-Rank Test to compare each model with DSnet, where a p-value < 0.05 indicates a significant difference between the two methods. It is worth noting that “DSnet (right)” refers to the exclusive training of the right hippocampal subfield segmentation branch of DSnet. Significant values are in italics.

		CA1-3	CA4/DG	SUB	Hippocampus
3D U-Net^40^	Mean (std)	3.86 (1.31)	4.67 (2.09)	3.39 (2.07)	8.21 (2.78)
	p-value	*0.10*	*0.77*	*0.78*	*0.12*
TransBTS^30^	Mean (std)	3.84 (1.70)	4.35 (1.98)	3.47 (1.56)	8.30 (2.42)
	p-value	*0.56*	*0.36*	*0.67*	*0.09*
AttU-Net^41^	Mean (std)	3.56 (1.27)	**4.34 (1.52)**	4.42 (3.45)	9.58 (3.03)
	p-value	*0.48*	*0.34*	*0.24*	*0.03*
DSnet (right)	Mean (std)	**3.36 (1.13)**	4.45 (1.82)	4.41 (3.73)	8.19 (2.75)
	p-value	*0.84*	*0.85*	*0.78*	*7.3e−3*
DSnet	Mean (std)	3.56 (1.45)	4.67 (1.56)	**3.25 (1.45)**	**7.68 (2.71)**

### Ablation experiment

We performed a series of ablation studies on the DSnet network, involving the removal of the CBAM structure, the elimination of the Transformer structure, and the omission of the hippocampus segmentation branch. All these experiments were conducted with consistent parameters, and the results are summarized in Table 3, which unmistakably demonstrate the positive impact of all three structures on the network’s accuracy. Notably, the absence of the CBAM structure has the most substantial influence, leading to an average accuracy reduction of 0.51%.

**Table 3:** Dice scores of segmentation results for the ablation study. “w/o CBAM” indicates the absence of the CBAM module, replaced by direct skip connections; “w/o Transformer” signifies the exclusion of the Transformer structure; “DSnet (right)” refers to the exclusive training of the right hippocampal subfield segmentation branch of DSnet. The superior results are highlighted in bold. Statistical analysis was conducted using the Wilcoxon Signed-Rank Test to compare each model with DSnet, where a p-value < 0.05 indicates a significant difference between the two methods. Significant values are in italics.

		CA1-3	CA4/DG	SUB	Hippocampus
DSnet (w/o CBAM)	Mean (std)	91.31 (1.2)	88.45 (1.6)	87.29 (2.0)	95.47 (1.0)
	p-value	*8.1e−4*	*1.9e−2*	*8.0e−4*	*6.7e−3*
DSnet (w/oTransformer)	Mean (std)	91.45 (1.2)	88.40 (1.8)	87.62 (1.9)	95.38 (0.8)
	p-value	*2.0e−2*	*4.2e−2*	*1.5e−2*	*3.4e−2*
DSnet (right)	Mean (std)	91.40 (1.2)	88.42 (1.9)	87.68 (1.9)	95.56 (0.6)
	p-value	*1.2e−3*	*3.2e−2*	*6.2e−2*	*2.3e−3*
DSnet	Mean (std)	**91.68 (1.2)**	**88.79 (1.6)**	**88.10 **(2.0)	**95.75 (0.6)**

In our method, the T1w images and T2w images are simultaneously input into the neural network model. T1w images typically exhibit good contrast between gray matter and white matter, clearly delineating structural boundaries. T2w images are particularly sensitive to tissues with higher water content, with fluid areas appearing brighter. We analyzed the impact of different imaging modalities on the segmentation accuracy of DSnet, with the experimental results shown in Table 4. The results indicate that using single-modality images as network input reduces segmentation accuracy. Specifically, using only T2w images, the average Dice coefficient for the hippocampus is 83.87%, which is 6.3% lower compared to using both modalities. This decrease in accuracy is due to the higher water content surrounding the hippocampus, resulting in less distinct contrast between the hippocampus and surrounding tissues compared to T1w images. Combining T1w and T2w images leverages the anatomical contrast of T1w and the water sensitivity of T2w, providing more comprehensive tissue information.

**Table 4:** Dice scores of segmentation results of dsnet using different modal images as inputs. The statistical analysis was performed using the Wilcoxon Signed-Rank Test, with T1w + T2w as the baseline. A p-value < 0.05 indicates a significant difference between the two methods. Significant values are in italics.

T1w	T2w		CA1-3	CA4/DG	SUB	Hippocampus
✓		Mean (std)	91.21 (1.3)	88.53 (1.9)	86.16 (2.3)	94.28 (0.9)
		p-value	*4.6e−3*	*5.8e−3*	*3.3e−6*	*5.2e−3*
	✓	Mean (std)	85.80 (5.6)	81.73 (4.7)	84.09 (6.6)	89.17 (2.8)
		p-value	*5.9e−8*	*6.0e−8*	*4.2e−7*	*3.4e−6*
✓	✓	Mean (std)	**91.68 **(1.2)	**88.79 **(1.6)	**88.10 **(2.0)	**95.75 (0.6)**

We also conducted ablation experiments on the application of the Transformer structure to different layers of the encoder. Since our images are three-dimensional, applying the Transformer to shallow network layers requires very high memory (exceeding our hardware limits), making it impractical for deployment. Therefore, we performed ablation experiments applying the Transformer to the fourth layer, the fifth layer, and both layers simultaneously. The experimental results are shown in Table 5. The results indicate that applying the Transformer only in the fifth layer (bottleneck layer) achieves higher segmentation accuracy. This is because the image features in the bottleneck layer have undergone multiple convolutions, containing rich high-level information, making them more suitable for global context interaction and extraction using the Transformer.

**Table 5:** Dice scores of segmentation results by applying the transformer to different layers of DSnet. The statistical analysis was performed using the Wilcoxon Signed-Rank Test, with the application of the Transformer in Layer 5 as the baseline, comparing it with other combination methods. A p-value < 0.05 indicates a significant difference between the two methods. Significant values are in italics.

Transformer			CA1-3	CA4/DG	SUB	Hippocampus
Layer 4	Layer 5					
✓		Mean (std)	91.08 (1.3)	88.18 (2.1)	87.05 (2.0)	95.11 (0.8)
		p-value	*3.2e−5*	*4.2e−3*	*1.6e−2*	*2.4e−3*
✓	✓	Mean (std)	91.18 (1.3)	88.32 (1.8)	87.36 (1.4)	95.48 (0.8)
		p-value	*5.6e−4*	*3.8e−3*	*3.1e−3*	*4.4e−4*
	✓	Mean (std)	**91.68 **(1.2)	**88.79 **(1.6)	**88.10 **(2.0)	**95.75 (0.6)**

## Discussion and conclusion

The integration of the transformer module into our network architecture significantly enhanced segmentation performance by capturing long-range dependencies and contextual information more effectively. Transformers are well-known for their ability to model global relationships within the data, which is particularly beneficial for tasks like image segmentation where context is crucial. Our results, as shown in Table 3, demonstrate a marked improvement in segmentation accuracy when the transformer is included. This improvement is statistically significant, as confirmed by the Wilcoxon Signed-Rank Test, indicating that the enhancement is not merely due to chance.

The CBAM contributed to performance gains by refining the feature maps through attention mechanisms. CBAM applies both spatial and channel-wise attention, allowing the network to prioritize the most informative parts of the input data. This selective focus on relevant features helps to improve the robustness and accuracy of segmentation. In our experiments, incorporating CBAM led to an additional increase in the Dice coefficient over the model with only the transformer, and this improvement was again statistically validated. This suggests that the attention mechanisms are effectively enhancing the network’s feature representation capabilities.

The dual-branch architecture leverages the principles of multi-task learning by having separate branches for different aspects of the segmentation task. This setup allows for more specialized and efficient feature extraction, which in turn enhances overall performance. Our findings indicate that the dual-branch model outperforms single-branch configurations, with improvements in both segmentation accuracy and consistency. The statistical analysis supports this observation. This indicates that the multi-task effect of the dual-branch architecture is a key factor in achieving better results.

To ensure that the observed improvements were not solely due to an increased number of parameters, we conducted additional experiments. We applied the transformer to different layers. As shown in Table 5, adding the transformer to the fourth layer, or to both the fourth and fifth layers simultaneously, resulted in significantly lower performance. This indicates that the improvements provided by our network architecture are not just due to an increase in parameters, but are intrinsic to the design improvements themselves.

In summary, we proposed DSnet, a meticulously designed dual-branch network architecture tailored to the precise segmentation of hippocampal subfields. This innovative structure incorporates a branch for hippocampus segmentation and another dedicated to hippocampal subfield segmentation, with the latter adaptively extracting image feature information from the former. DSnet further integrates the CBAM structure at each encoder level, replacing traditional skip connections, thus empowering the decoder to more effectively harmonize information extracted at varying encoder levels. Simultaneously, we introduced a Transformer structure at the network’s foundation for robust contextual feature extraction. Empirical results demonstrate that our method performs comparably to mainstream state-of-the-art segmentation techniques, highlighting its effectiveness in hippocampal subfield segmentation.

However, we acknowledge that the performance of our method is intrinsically linked to the quality and quantity of the dataset employed. Presently, our experiments have been conducted using a specific dataset, which may not comprehensively represent the full spectrum of variability encountered in clinical settings. Our forthcoming research endeavors will prioritize collaboration with medical professionals for extensive clinical validation. This collaborative effort will serve as a critical step toward ensuring the accuracy and reliability of our approach in the complex and dynamic landscape of real-world healthcare settings. Through these concerted efforts, we aim to fortify the foundations of our method and enhance its applicability as a valuable tool in the realm of medical image analysis.

## Data Availability

The data supporting the findings of this study are publicly available (https://www.nitrc.org/projects/mni-hisub25).
